# Microbial Community Interactions Are Sensitive to Small Changes in Temperature

**DOI:** 10.3389/fmicb.2021.672910

**Published:** 2021-05-21

**Authors:** Emil Burman, Johan Bengtsson-Palme

**Affiliations:** ^1^Department of Infectious Diseases, Institute of Biomedicine, The Sahlgrenska Academy, University of Gothenburg, Gothenburg, Sweden; ^2^Centre for Antibiotic Resistance Research (CARe), University of Gothenburg, Gothenburg, Sweden

**Keywords:** microbial communities, biofilm, THOR, temperature, community interactions

## Abstract

Microbial communities are essential for human and environmental health, often forming complex interaction networks responsible for driving ecosystem processes affecting their local environment and their hosts. Disturbances of these communities can lead to loss of interactions and thereby important ecosystem functionality. The research on what drives interactions in microbial communities is still in its infancy, and much information has been gained from the study of model communities. One purpose of using these model microbial communities is that they can be cultured under controlled conditions. Yet, it is not well known how fluctuations of abiotic factors such as temperature affect their interaction networks. In this work, we have studied the effect of temperature on interactions between the members of the model community THOR, which consists of three bacterial species: *Pseudomonas koreensis*, *Flavobacterium johnsoniae*, and *Bacillus cereus*. Our results show that the community-intrinsic properties resulting from their interspecies interactions are highly dependent on incubation temperature. We also found that THOR biofilms had remarkably different abundances of their members when grown at 11, 18, and 25°C. The results suggest that the sensitivity of community interactions to changes in temperature is influenced, but not completely dictated, by different growth rates of the individual members at different temperatures. Our findings likely extend to other microbial communities and environmental parameters. Thus, temperature could affect community stability and may influence diverse processes including soil productivity, bioprocessing, and disease suppression. Moreover, to establish reproducibility between laboratories working with microbial model communities, it is crucial to ensure experimental stability, including carefully managed temperature conditions.

## Introduction

Microbial communities are assemblages of microbes that co-inhabit the same environment. They are ubiquitous among all biotopes and are present on, and in, all macroorganisms (Finlay and Clarke, 1999). The health of microbial ecosystems affects many different aspects of human life, including, for example, securing the food supply, where symbiotic relationships between many of our food crops and the microbes in the rhizosphere are crucial for optimal crop yields (Zorner et al., 2018). Microbial communities also affect human health more directly, through establishment of symbiotic and commensal bacteria in, for example, the human gut. These communities have intricate relationships between themselves and with the host, extending as far as being able to modulate the availability of neurotransmitters to the host (Strandwitz, 2018). Microbial communities have also been shown to block the establishment of pathogens in the gut microbiome, thus acting as a barrier that prevents potential pathogenic bacteria to colonize the lumen of the gut (Kamada et al., 2013).

While microbes typically have high reproduction rates, allowing for analysis of microbial communities with high throughput, many natural communities have networks of often undefined interactions of antagonisms and synergisms, making these interactions strenuous to analyze *in vitro*. Model microbial communities have been suggested as a remedy to this complexity, permitting elucidation of the mechanisms behind community interactions at the genetic and molecular level (Chevrette et al., 2019; Bengtsson-Palme, 2020). These simplified model microbial communities are often more suitable than full-scale complex communities for *in vitro* dissection, thanks to that many of the member interactions are already defined. Many model microbial communities have standardized protocols of analysis readily available, which allows for laboratory analysis without establishing working conditions for the community (Blasche et al., 2017).

Environmental factors define the biological boundaries imposed upon microbial communities, and thus the viability and phenotypic expression of the community is restricted by these factors (Else et al., 2003). Such environmental factors include UV radiation, pH, salinity, barometric pressure, nutrient availability, presence of inhibitory substances, and temperature (Yin et al., 2019). For example, in *Pseudomonas putida*, it has been shown that production of biofilm is increased at 10°C compared to 25°C (Morimatsu et al., 2012). Furthermore, short-term heat shock treatment of *Pseudomonas aeruginosa* cultures reduces the amount of culturable biofilm by up to six orders of magnitude (O’Toole et al., 2015). Therefore, it is likely that a change in growth temperature could affect the viability, longevity and interactions of microbial communities. Interestingly, this has been shown in several instances. In microbial communities isolated from hot springs in Yellowstone National Park, a relatively small change in temperature at thermophilic ranges strongly affected community interactions (Hunt et al., 2018). Similarly, in the microbial communities in hotsprings in Tenchong, China, both iron availability and temperature affected community interactions in thermophilic conditions (He et al., 2021). Finally, microbial anaerobic communities have been shown to have differences in community interactions measured in terms of methane production in a relatively large span of temperatures (Lin et al., 2016). Taken together, these communities and the effects of temperature changes on their interactions suggest that such relationships may be common, also in microbial communities that do not originate from thermophilic or anaerobic hotspots, such as, those in soil. This would have several implications; for example, many microbial communities may only have a small temperature span where community interactions take place. Thus, depending on the temperature the community is grown under, one could get different degrees of community interaction, resulting in that some community interactions may not be identified despite being highly important for community functions in other conditions.

This work utilizes the microbial model community THOR—The Hitchhikers of the Rhizosphere—to analyze how temperature influences the stability of interactions in microbial communities. THOR consists of three bacterial species, *Pseudomonas koreensis*, *Flavobacterium johnsoniae*, and *Bacillus cereus* (Lozano et al., 2019). They belong to the dominant phyla in the rhizosphere: Proteobacteria, Bacteroidetes, and the Firmicutes, respectively, and the three species have been shown to act as a community through both antagonisms and synergisms.

When the three species of THOR are grown together and interact with each other, they show a pattern of biofilm production in which all monocultures and co-cultures produce lower amounts of biofilm than what is produced when all three members are grown together, in what we will refer to as a “Triple Culture.” Following the definition by Madsen et al. (2018), *community-intrinsic properties* are “properties of bacteria that only arise in the context of a community, not in isolation.” In the conditions THOR is grown under in this study, only *P. koreensis* produces any significant amount of biofilm, while *B. cereus* and *F. johnsoniae* do not. Our null model for this interaction experiment is thus that adding either *B. cereus* or *F. johnsoniae* to *P. koreensis* would have little impact on biofilm formation, unless there is an interaction taking place between the species. Moreover, if there would be a competitive interaction between the species, it would be more likely to show as less biofilm formation (Bengtsson-Palme, 2020). However, when the three THOR members are grown together, they form up to three times more biofilm than what *P. koreensis* does on its own. Co-culturing of *P. koreensis* together with *B. cereus* or *F. johnsoniae* leads to increased biofilm formation compared to when *P. koreensis* is grown alone, and the triple culture results in a denser biofilm with longer longevity compared to culturing either monocultures or co-cultures of the THOR constituents (Lozano et al., 2019). This increased biofilm production above the expected baseline for *P. koreensis* does not conform to the null hypothesis (and also not to the most simplistic competition hypothesis), and we thus, based on the definition by Madsen et al. (2018), consider the increased biofilm when the three species are grown together to be (1) a community-intrinsic property, and (2) likely to be the result of interactions between the members of the community.

The community-intrinsic biofilm production of THOR allows for screening of individual factors that disrupt the community interactions, as any disturbance to any of the community members will be reflected in the total production of biofilm by the community. This allows us to use total biofilm production as a reporter of community behavior in THOR. Thus, in contrast with previous studies on complex natural microbial communities and how their interactions are affected by changes in temperature, we will in this study be able to link, e.g., growth rates of the individual community members to changes in interaction patterns, providing more direct explanations of community-intrinsic properties (Bengtsson-Palme, 2020).

This work aims to systematically analyze the effect of small changes in temperature on the biofilm production of THOR, and whether disturbances to the community behaviors are the products of differential effects on growth of the different members at different temperatures. Such disturbances could have major implications on community stability thus affecting processes involved in, for example, soil productivity, bioprocessing, and disease suppression. Moreover, if interactions are easily disturbed by small changes to abiotic factors such as temperature, it would be curial to establish reproducible conditions between laboratories working with microbial model communities.

## Materials and Methods

### Bacterial Strains and Culture Conditions

The THOR members—*P. koreensis* CI12, *F. johnsoniae* UW101, and *B. cereus* UW85 (Table 1)—were obtained from the lab of Prof. Jo Handelsman (University of Wisconsin-Madison, WI, United States) and subsequently stored in LB (Luria Bertani) broth containing glycerol (15%) at −80°C (long term) or −20°C (short term). Plates were created by re-streaking for single colonies from the freezing cultures (24 h, 28°C) and isolates were then re-streaked once again for single colonies onto a new LB plate and incubated (24 h, 28°C). Plates were kept in a fridge (4°C) for a maximum of 1 week. Broth cultures were propagated by inoculating a single colony from a plate into Tryptic Soy Broth (TSB) at 50% strength and incubating it at 28°C overnight (14–18 h, 150 rpm).

**TABLE 1:** The members of THOR with their corresponding numbers and source publications.

Species	Strain	Source
*Pseudomonas koreensis*	CI12	Peterson et al., 2006
*Flavobacterium johnsoniae*	UW101	McBride and Braun, 2004
*Bacillus cereus*	UW85	Handelsman et al., 1990

### 96-Well Crystal Violet Assay

The ability of THOR to form biofilm on surfaces was quantified using a 96-well plate crystal violet assay. Overnight cultures of each of the member strains were diluted in 10% TSB to species-specific concentrations. The final optical density at 600 nm (OD_600_) in each well was 0.004 for *P. koreensis*, and 0.001 for *F. johnsoniae* and *B. cereus*. This corresponds to around 10^4^ CFUs per well for *P. koreensis* and *B. cereus* and just below 10^5^ CFUs per well for *F. johnsoniae* (Supplementary Figure 1). Each species was then transferred to the plate for a total well volume of 200 μL. The plate was incubated statically for 24 h at different temperatures (9, 11, 13, 15, 17, 18, 19, 20, 21, 23, 25, or 28°C), and OD_600_ was measured using a Fluo-star Omega Microplate reader. The growth medium was discarded, and the wells were washed with PBS (phosphate buffered saline; 200 μL) three times. The plate was stained with crystal violet (200 μL, 0.1%) for 60 min, washed with PBS (400 μL) once, and then washed with PBS (200 μL, twice). The stain was released using acetic acid (33%, 200 μL) for 30 min, and absorbance (λ = 595) was recorded using a FLUOstar Omega Microplate reader. From the obtained values, the background noise was subtracted, and the resulting values were divided by the background-corrected values for *P. koreensis* in order to relate the amount of biofilm produced by the community to that of *P. koreensis* alone, as *P. koreensis* is the main biofilm former in the community (Supplementary Figure 2). This analysis was performed using eight technical replicates for each biological replicate (*n* = 4). For each biological replicate, the median of the technical replicates was used for all calculations to reduce the impact of individual technical replicates influencing the quantification of the biofilm.

### Colony-Forming Units Isolated From THOR Biofilms

The abundances of the three THOR members at different incubation temperatures were quantified using a selective plating assay. Overnight cultures of each member of the THOR microbial community were diluted (10% TSB) to an OD_600_ of 0.004 for *P. koreensis*, 0.001 for *F. johnsoniae*, and 0.001 for *B. cereus*. The THOR members were then transferred (200 μL) to the microtiter plate and incubated at 11, 18, or 25°C for 24 h. OD_600_ was measured using a Fluo-star Omega Microplate reader. The medium was discarded, and the wells were washed (PBS, 200 μL) three times. PBS (200 μL) was added and the biofilms were incubated at room temperature (2 h). The walls of each well were scraped up and down with a sterile pipette tip to harvest the surface-attached biofilm. The harvested liquid was transferred to a 96-well titer plate and was 10-fold serially diluted seven times with PBS. The dilution series was then plated (5 μL/spot) onto LB plates with selective agents specific to each THOR member. The selective plates contained the following antibacterial agents and were incubated as followed, Polymyxin B (5 μg/mL) for *B. cereus* (16 h, 28°C), Gentamycin (10 μg/mL) for *F. johnsoniae* (48 h, 28°C) and Erythromycin (3 μg/mL), Ampicillin (100 μg/mL), and Carbenicillin (125 μg/mL) for *P. koreensis* (20 h, 28°C). The colony-forming units (CFUs) were counted in the dilution spot where the highest number of colonies could be reasonably counted by visual inspection, for each respective dilution series. The counts were then entered into a Microsoft Excel spreadsheet, where Students two-tailed *t*-tests and visualization of data were performed. This analysis was performed with five technical replicates for each biological replicate (*n* = 6).

## Results

**FIGURE 1:**
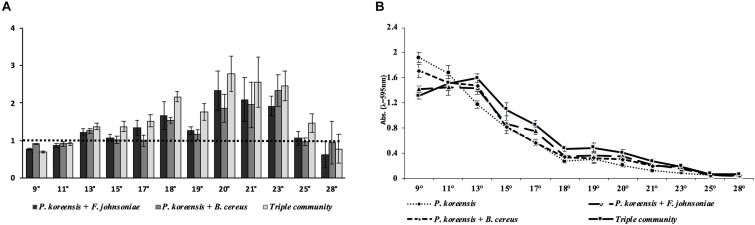
Temperature-dependent biofilm production in THOR. **(A)** Average amount of biofilm produced by biofilm-producing co- and triple cultures of the THOR members relative to the amount of biofilm produced by the monoculture of *Pseudomonas koreensis*, which is represented by the dotted line. The *y*-axis scale is shown as ratios of the *Pseudomonas koreensis* monoculture biofilm production. **(B)** Total amount of biofilm produced by THOR members. Temperatures are given in °C. The error bars represent the standard error of the mean across four biological replicates.

We quantified the total amount of surface-attached biofilm produced by the THOR model microbial community using a crystal violet assay, in which the community was incubated at temperatures flanking room temperature and then compared to the amount of biofilm formed by *P. koreensis* alone (Figure 1A).

We observed that at 25°C the described ability of THOR to produce biofilm via a gradual increased pattern was completely lost (Figure 1A). In contrast, at 18°C a clear gradual increase in biofilm production was observed, in which both the co-cultures and the triple community showed higher-than-expected biofilm production compared to the amount of biofilm produced by the individual community members alone. Specifically, the addition of *F. johnsoniae* increased biofilm formation by 66% and addition of *B. cereus* increased it by 53%, compared to the monoculture of *P. koreensis*. In the triple community, biofilm production increased by 117% at 18°C (Figure 1A). In addition, we observed the largest amount of biofilm produced across all tested temperatures at 9°C (Figure 1B). At this temperature, the relative biofilm production of the co-culture of *P. koreensis* and *F. johnsoniae* was 26% lower, and for the co-culture with *B. cereus*, it was down 11% compared to the biofilm formed by *P. koreensis* alone. In the triple community, biofilm production was 32% lower than for *P. koreensis* grown alone. Our results show that the amount of biofilm produced, and which co-cultures produce the most biofilm, is dictated by the temperature at which the community is grown.

We furthermore observed that incubation temperature strongly affected the amount of biofilm produced by THOR. This effect occurred across all the conditions; the monoculture of *P. koreensis*, the co-cultures of *P. koreensis* with *F. johnsoniae* and *B. cereus*, and in the triple community (Figure 1B). Also, we observed that at 25 and 28°C, the ability of THOR to produce biofilm was almost completely decimated.

**FIGURE 2:**
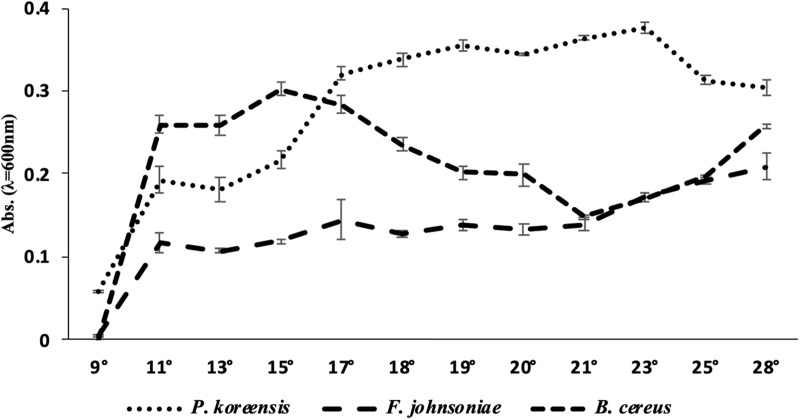
Growth (OD_600_) after 24 h for the THOR members grown alone at different temperatures. Temperatures are shown in °C. The error bars represent the standard error of the mean across the four biological replicates. Note that the growth has been measured when the strains have been grown alone, without the other THOR members.

Once we had observed that the amount of biofilm produced by THOR was dependent on growth temperature, we turned to analyze the planktonic growth of the THOR members at the temperatures flanking room temperature, to investigate if this effect was directly related to different growth rates of the community members. We observed that the individual growth of the different THOR members in the absence of the other species was highly dependent on temperature (Figure 2). For *B. cereus*, we found that at 11°C, the OD_600_ was 0.257, but that the OD_600_ increased to 0.302 at 15°C. The growth then decreased with increasing temperature until 21°C, where it started increasing again up to the final temperature of 28°C, where the OD_600_ was 0.258. For *P. koreensis*, we observed that the OD_600_ at 11°C was 0.193 which increased to 0.32 at 17°C, where the growth seemed to reach a plateau with only modest changes in OD_600_. At 28°C, the OD_600_ for *P. koreensis* was 0.303. For *F. johnsoniae*, we observed an OD_600_ at 11°C of 0.116, which then slowly increased with higher temperature, with its largest OD_600_ being at 28°C (0.209). At 9°C, all three species grew quite poorly, with OD_600_ below 0.1.

Since the OD_600_ was observed to be strongly dependent on temperature, we postulated that similar differences in growth could also be observed in the biofilms. To investigate that, THOR was grown at 11, 18, and 25°C, which were selected as representative of the relative growth patterns across temperatures, and biofilms were harvested for CFU counting.

**FIGURE 3:**
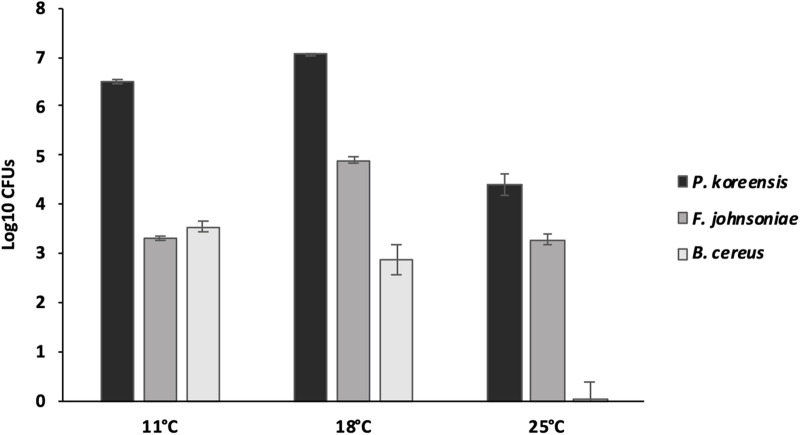
Number of biofilm CFUs per well for the THOR members grown at different temperatures. Averages across six biological replicates are shown, and the error bars represent the standard error of the mean.

By selectively isolating the THOR community members from the biofilms, we discovered differences in abundance of the constituent members when the community was grown under different temperatures (Figure 3). For *P. koreensis* at 11°C, 3.19 × 10^6^ CFUs were observed, at 18°C this had increased to 1.13 × 10^7^, while at 25°C only 4.50 × 10^4^ CFUs were detected. *F. johnsoniae* produced 2.03 × 10^3^ CFUs at 11°C, 8.26 × 10^4^ CFUs at 18°C, and 2.20 × 10^3^ at 25°C. For *B. cereus*, 3.87 × 10^3^ CFUs were observed at 11°C, at 18°C 1.77 × 10^3^ CFUs, and at 25°C a dramatic drop of CFUs was noted, resulting in that live bacteria could only be detected in one out of five replicates. Clearly, *P. koreensis* is the dominant species in the biofilm, with the other two species having at least two orders of magnitude lower CFU counts. The large differences between the number of cells belonging to *P. koreensis* and the two other species, combined with the fact that the other two members still have a major impact on biofilm formation (Figure 1), suggest that interactions are taking place between the community members. Moreover, the ability to participate in interactions in the biofilms was affected by temperature; however, except in the case of the decrease of *B. cereus* at 25°C, this could not directly be related to differential abundance of the three species at different temperatures.

## Discussion

Community-intrinsic properties arise due to interactions between microbes in communities, and are defined as deviations from what would be expected based on how individual community members behave when cultured individually (Madsen et al., 2018). The increased biofilm formation of THOR compared to the biofilm produced its members when grown alone can be considered such a community-intrinsic property resulting from interactions between the members of the community. In this work, we show that this increased biofilm formation is not simply the result of the addition of more cells to the biofilm by *B. cereus* or *F. johnsoniae*. Instead, the CFU results show that *P. koreensis* still dominates the biofilm by orders of magnitude. This suggests that interactions take place between the THOR members, and that the added biofilm is not simply a by-product of that *B. cereus* and/or *F. johnsoniae* are able to colonize a surface that *P. koreensis* has made available to them.

Furthermore, this work shows that community-intrinsic properties resulting from interactions in the THOR model community are highly dependent on the temperature at which the community is grown. The observed differences in biofilm production induced by small changes in temperature seem to be partially related to changes in relative growth of the individual microbial community members, both in liquid culture and in biofilms, although different growth rates cannot fully explain the patterns of biofilm formation at different temperatures. Having said that, a change in growth rate resulting from a change in temperature could be discriminatory against individual members of the community, which seems to the case with *B. cereus* and *P. koreensis* in our experiments. This will alter the relative abundances of the member species in the community, and our results indicate that this in turn affects biofilm production, since it is largely governed by the abundance of certain bacteria in a given setting (Davies et al., 1998). The described temperature dependence of THOR biofilm formation could partially be explained by alterations of growth rates of the individual THOR members at different temperatures. For *P. koreensis*, we observed a CFU difference on the order of ∼10^3^ between the temperature points of 18 and 25°C and a decrease of biofilm production from 0.41 to 0.06 in the crystal violet assay. Since *P. koreensis* is the main producer of biofilm in this microbial model community (Lozano et al., 2019; Supplementary Figure 2), its lower abundance in the biofilm explains why the total amount of biofilm produced decreased at this temperature. However, these changes in the biofilm did not correspond to lower OD_600_ absorbance (an indicator for the degree of planktonic growth) at this temperature for *P. koreensis*. Notably, an even larger change in absolute biofilm production happened between 11 and 18°C, where biofilm readout decreased from 1.51 to 0.41 despite only modest changes in number of *P. koreensis* CFUs between those two temperatures. This suggests that while growth rates are important for the changes in biofilm formation in THOR at different temperatures, it cannot alone explain these alterations, and thus community interactions specifically are likely to be perturbed by the temperature differences. Interestingly, there was a pronounced difference in the abundance of *F. johnsoniae* in the biofilms at 11 and 18°C, suggesting that the ratios between the different members are also of importance for the community-intrinsic properties of THOR. Furthermore, at 25°C *B. cereus* could no longer be detected in the community in most replicates, suggesting that it may potentially be completely eradicated. At the same time, we did not observe a strong reduction of *F. johnsoniae*, which is notable as *B. cereus* has been described to have a protective effect on *F. johnsoniae* inhibition by *P. koreensis*. However, the protective effect of *B. cereus* against *P. koreensis* has been shown to be highly context dependent and has mainly been observed in root exudate medium (Lozano et al., 2019). This work was performed using 10% TSB, and thus the inhibitory effects of *P. koreensis* on *F. johnsoniae* might be lower, or missing entirely.

Our observation that the biofilm production community-intrinsic property of THOR is strongly influenced by temperature suggests the existence of a temperature at which maximum community interaction occurs. Based on our data, we believe that this temperature is 18°C, which is where the biofilm formation differences between the mono-, co-, and triple cultures are most apparent and stable between experiments. Furthermore, a small decrease in temperature drastically increased the amount of biofilm produced in all biofilm-forming species combinations. Nevertheless, by decreasing the temperature, the community-intrinsic biofilm production behavior of THOR became less apparent or disappeared completely. Similarly, a relatively small increase in temperature to 25°C almost completely decimates the amount of biofilm produced by THOR. Such small changes either up or down in temperature are conceivable when incubating the community in a thermally unregulated environment. Thus, a high degree of temperature control is crucial between experiments, particularly when reproducing results across different laboratories, equipment, and personnel. We also observed that biofilm production in the THOR microbial community became highly variable between experiments at temperatures above 20°C. This is largely due to the low amount of total biofilm produced at these temperatures. Notably, at these temperatures, the crystal violet readout is close to the detection limit of the plate reader. This means that the signal-to-noise ratio at the higher temperatures is substantially higher than in the lower end of the temperature spectrum. Together, these results highlight the need for standards and transparency in research on microbial model communities (Bengtsson-Palme, 2020).

Specifically for THOR, we along these lines recommend incubation at 18°C, for example, in a cold incubator, rather than at room temperature, especially if room temperature cannot be precisely controlled. While we in this study have focused on the effects of temperature on community interactions, we suspect that individual members of model communities including, but not limited to, THOR and the interactions between them might be similarly influenced by changes of other environmental factors, including pH and nutrient availability. Furthermore, disruptive factors that discriminate against single members of the community are not unique to THOR. Rather, this is a problem that would be shared with other microbial communities as well, both in natural settings and in microbial community models. This has important implications for research employing model microbial communities, since only a few of these model communities have been elucidated for community behaviors outside of specific culturing conditions they were first contrived under. Importantly, this may severely limit our view of interactions between microbes in communities to specific laboratory settings, throwing doubt on the validity of extrapolation from these results. While we would welcome clearly defined growth conditions for every microbial community model, we hope that more research will be performed on the stability of observed community-intrinsic behaviors in the face of variable environmental conditions, such as temperature, pH, nutrient availability, and initial inoculum size.

In this work, we have shown that the amount of biofilm produced by *P. koreensis* increases with a decrease in temperature. This is true for the mono-, co-, and triple cultures. Interestingly, the community-intrinsic biofilm production of THOR was reversed at 11°C, such that the *P. koreensis* monoculture produced more biofilm than the co-cultures and triple cultures at this temperature. One potential explanation to the behavior described above could be that growth patterns are time shifted, leading to that the peak of biofilm formation may be delayed at lower temperatures. However, this is unlikely to be the only factor influencing the changes in community behavior, as this work has demonstrated that the relative abundances of the members change with temperature. In the psychrophilic soil bacterium *Pseudomonas mandelii*, it has been shown that a decrease from 25 to 5°C increases the amount of biofilm produced sixfold in LB broth (Vásquez-Ponce et al., 2017). Similarly, in *P. putida*, another soil-living pseudomonad, it has been shown that culturing at 5°C increases yield and longevity of the biofilm compared to culturing at 30°C (Morimatsu et al., 2012). Thus, it is not surprising that soil-residing pseudomonads produce a larger amount of biofilm at lower temperatures than at higher temperatures. This suggests that biofilm production might be a common mechanism that soil bacteria use to cope with lower temperatures.

Temperature sensitivity of interactions between microbes has also been discovered in many different natural communities. In soil, short-term extreme heat shock (>100°C) has been shown to cause a long-term reduced growth of the treated microbial communities persisting for multiple generations (Barreiro et al., 2020). More moderate long-term alterations of growth temperature in Alpine soils have shown that these soil communities have a growth optimum between 27 and 30°C and that the community structure was substantially altered, in accordance with the findings in this study. However, the legacy of the soil source seems to dictate the ability of the community to respond to a higher growth temperature (Donhauser et al., 2020). Furthermore, interspecies interactions have been connected to temperature, with a decrease in interactions with an increase in temperature in soil microbiomes (García et al., 2018). In marine microbial communities, it has been shown that an increase in temperature by 3°C, approximately corresponding to expected effects global warning, significantly alters the community composition (Wang et al., 2021). In addition, with similar changes in temperature, we observed drastically reduced interspecies interactions due to shifts in community composition, highlighting the potential of global warming to disrupt interaction networks in microbial communities. Disturbances to these networks can lead to loss of important ecosystem functionality and stability. These described effects on community composition, growth, and interactions demonstrate that temperature affects community interactions not only in THOR, but likely in almost every natural microbial community as well. Furthermore, not only temperature has been found to influence microbial community growth and interactions, but also pH (Wang et al., 2021) and nutrient availability (Mello et al., 2016). Taken together, these studies show that seemingly innocuous factors can greatly affect how microbial communities behave. In this study, we link these changes in behavior to specific differences in growth between the members of a model community, showing the power offered by such models to explain phenomena occurring in natural settings (Bengtsson-Palme, 2020).

In conclusion, we have in this study determined that the model microbial community THOR is sensitive to small changes in temperature, and that the changes in community-intrinsic properties are partially dependent on the relative abundances of the constituents in the biofilm, which in turn are governed by different growth rates of the THOR members at different temperatures. These results have important implications both for standardization of research on model microbial communities and for our understanding of microbial interactive behavior writ large. Altered interactions in microbial communities have the potential to affect diverse processes including soil productivity, bioprocessing, and disease suppression and thus have implications for both human and environmental health.

## Data Availability Statement

The raw data supporting the conclusions of this article will be made available by the authors, without undue reservation.

## Author Contributions

EB performed laboratory analyses. EB and JB-P designed the project, interpreted the data, wrote the manuscript, and approved the final version. JB-P provided funding for the project. Both authors contributed to the article and approved the submitted version.

## Conflict of Interest

The authors declare that the research was conducted in the absence of any commercial or financial relationships that could be construed as a potential conflict of interest.
