# Supplementary material for: Microbial Community Interactions Are Sensitive to Small Changes in Temperature
Source: Front Microbiol. 2021 May 21;12:672910. doi: 10.3389/fmicb.2021.672910 (PMC8175644; doi:10.3389/fmicb.2021.672910)
Supplement: Supplementary file 1 [file Image_1.pdf]

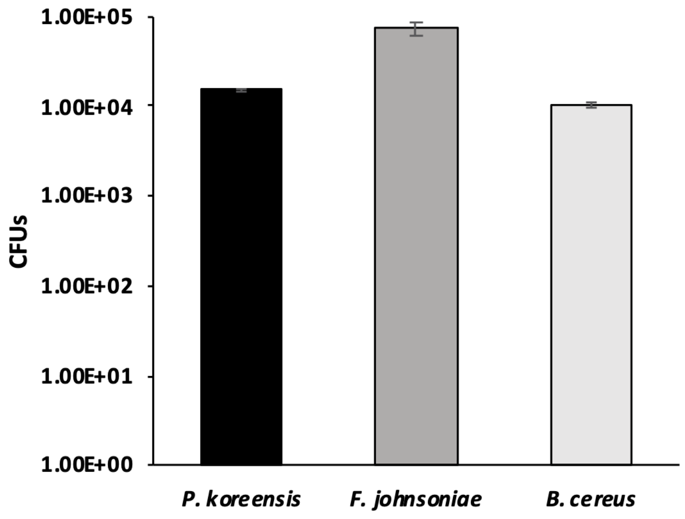

Supplementary figure 1. The #CFU of the different THOR member at moment of inoculation. Shown here, are the seed size across four biological replicates with the error bars being the standard error of the mean.

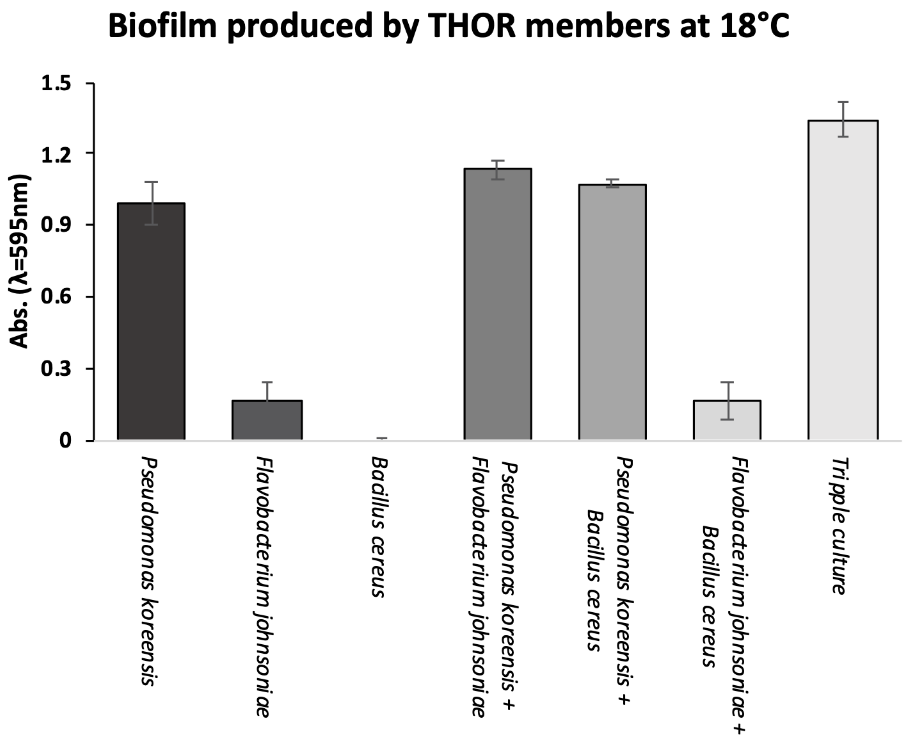

Supplementary figure 2. The amount of biofilm produced by THOR at 18 degrees across four biological replicates. The y axis unit is absorbance (595nm). This replicate perfectly shows how the addition of each additional member adds to the total amount of biofilm produced by the community.
